# Adjuvant radiotherapy treatment of chordoid glioma: a case report with a literature review

**DOI:** 10.3389/fonc.2025.1680822

**Published:** 2025-11-17

**Authors:** Francesco Cuccia, Salvatore D’Alessandro, Marco Messina, Giovanni Tringali, Giuseppe Craparo, Livio Blasi, Francesco Azzarello, Giuseppe Carruba, Giuseppe Ferrera

**Affiliations:** 1 Radiation Oncology, Azienda ospedaliera di Riferimento Nazionale e di Alta Specializzazione (ARNAS) Civico Hospital, Palermo, Italy; 2 Medical Oncology, ARNAS Civico Hospital, Palermo, Italy; 3 Neurosurgery Unit, ARNAS Civico Hospital, Palermo, Italy; 4 Neuroradiology Unit, ARNAS Civico Hospital, Palermo, Italy; 5 Medical Physics, ARNAS Civico Hospital, Palermo, Italy; 6 Division of Internationalization and Health Research (SIRS), ARNAS Civico Hospital, Palermo, Italy

**Keywords:** chordoid glioma, radiotherapy, adjuvant treatment, rare tumors, CNS tumors

## Abstract

**Background:**

Chordoid glioma is a rare World Health Organization (WHO) Grade II brain tumor located near the third ventricle. Fewer than 100 cases have been reported in the literature, and surgery represents the main treatment option. Due to its typical location, complete surgical resection is uncommon, and the role of adjuvant radiotherapy remains controversial.

**Methods:**

Based on a case report, we performed a literature review focused on the potential role of adjuvant radiotherapy for chordoid glioma. We summarized data on patient characteristics, surgical approach and extent of resection, radiotherapy technique and dose, and clinical outcomes.

**Results:**

A total of 18 patients from 14 studies were identified. Stereotactic treatments were used more frequently than conventional external beam radiotherapy, with doses ranging from 11.5–18 Gy and 45–59.4 Gy, respectively, and a median local control time of 26 months. Five patients developed disease recurrence after a median of 22.4 months. In the present case, the patient received adjuvant conventional radiotherapy (59.4 Gy/33 fractions) and remains alive after 12 months of follow-up with no major side effects.

**Conclusions:**

From the available evidence, modern radiotherapy may be considered a therapeutic tool that combines less invasive surgical procedures with improved local control, potentially reducing the risk of severe postoperative complications. Larger studies with longer follow-up periods are needed.

## 1 Introduction

In 1998, Brat et al. first described chordoid glioma (CG) as a brain tumor typically located near the third ventricle, characterized histologically by glial fibrillary acidic protein (GFAP)-positive tumor cells surrounded by a myxoid matrix (1, 2).

The most recent World Health Organization (WHO, 2021) classification of central nervous system (CNS) tumors lists CG as an astrocytic Grade II tumor due to its relatively slow growth pattern (3). Nonetheless, its peculiar location is closely associated with the sudden onset of neurological symptoms, including hydrocephalus (due to cerebrospinal fluid obstruction), headache, behavioral disorders, visual impairment, ataxia, and memory issues (4).

From a radiological perspective, CG usually appears on MRI as a well-defined, midline, suprasellar, contrast-enhancing mass, with infrequent invasion of the surrounding brain tissue (5). Maximal safe resection remains the mainstay of treatment, although the critical location often precludes complete resection, resulting in a higher risk of disease relapse or the need for adjuvant treatment. Moreover, the postoperative course is frequently complicated by endocrine and metabolic sequelae, such as hypothalamic dysfunction, diabetes, and other endocrinological disorders (6–8).

Fewer than 100 cases have been reported in the literature, with variable outcomes and limited understanding of the disease behavior in terms of recurrence patterns, adjuvant treatment, and prognosis. Due to the rarity of the disease, there is currently no standardized treatment approach. In particular, no consensus has been established regarding the optimal dose and fractionation schedule for radiotherapy.

In this article, we describe the clinical course of a young adult woman with a third ventricle CG treated with surgery followed by adjuvant radiotherapy and discuss this case in the context of published literature, with a specific focus on the potential role of adjuvant radiotherapy.

## 2 Methods

A PubMed and Embase literature search was conducted in December 2024 using the following MeSH terms: “chordoid” AND “glioma” AND “radiotherapy,” covering publications from 2000 to 2024. Additional hand searching of reference lists from the retrieved articles was also performed.

For this review, all articles published in English were included. Reviews or papers focusing exclusively on the histopathological features of the tumor, without reporting clinical outcomes, were excluded. Case reports and case series were appraised using the JBI Critical Appraisal Checklist (28).

Two independent reviewers (FC and SDA) performed the data collection, and a third reviewer (MM) was involved in cases of disagreement. Data were extracted on baseline patient characteristics (age, sex, presenting symptoms, and tumor size), surgical approach and extent of resection, radiotherapy technique and dose (where available), and clinical outcomes. Because of the rarity of the disease, studies without radiotherapy details were also included.

## 3 Results

### 3.1 Case description

A 42-year-old woman was admitted to our hospital due to the onset of severe headache, ataxia, visual impairment, and speech disorders.

Her medical history was positive for smoking, overweight, Raynaud syndrome, hypertension, and a previous cervical conization for a high-grade squamous intraepithelial lesion.

**Figure 1:**
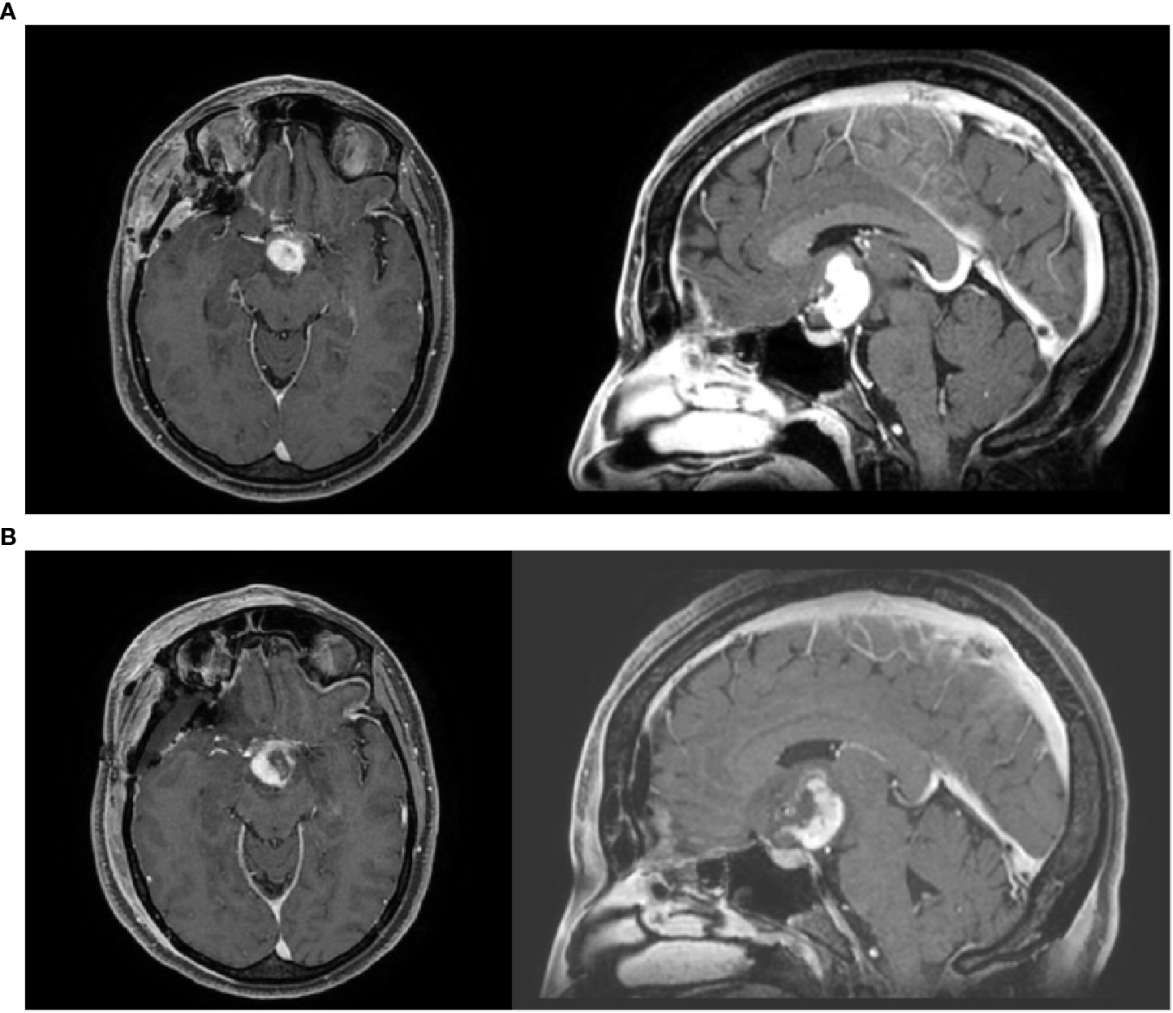
**(A, B)** Pre-operative and post-operative MRI imaging.

A contrast-enhanced brain computed tomography (CT) scan revealed an ovoid solid mass measuring 2.9 × 2.6 cm. These findings were confirmed on contrast-enhanced brain MRI, which showed a homogeneously enhancing, polylobulated mass of 2.7 × 2.4 cm in the hypothalamic–chiasmatic region, causing compression of the left optic nerve, which appeared enlarged in the prechiasmatic portion. Moreover, bilateral involvement of the optic tracts was observed, along with anterior obstruction of the third ventricle and bilateral Monro foramina, although without hydrocephalus (**Figure 1A**).

**Figure 2:**
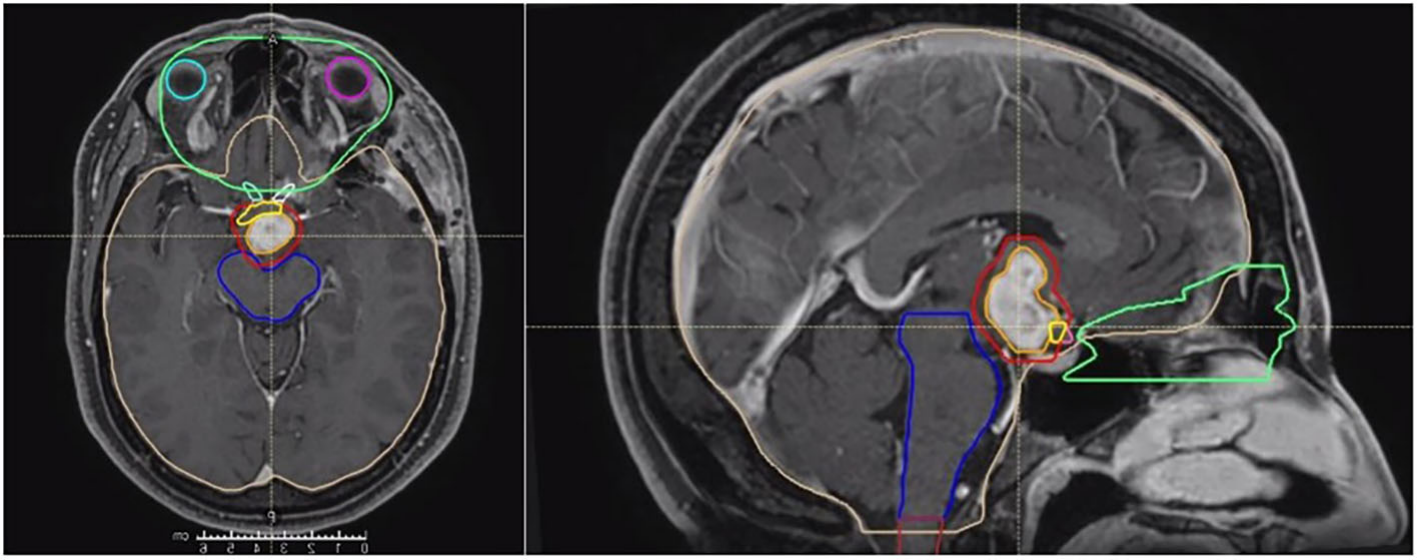
MRI imaging after surgery.

In January 2024, the patient underwent surgical excision of the tumor via a pterional approach. Histological examination revealed strong positivity for GFAP, synaptophysin, and thyroid transcription factor 1 (TTF-1), with focal expression of CKPan and a Ki-67 index of 2%. These findings confirmed a diagnosis of WHO Grade II chordoid glioma (**Figure 2**).

One month later, postoperative MRI revealed residual or relapsed disease measuring 1.9 × 2.7 cm. After multidisciplinary discussion, the patient was considered a candidate for adjuvant radiotherapy (**Figure 1B**).

At the initial evaluation in the Radiotherapy Department, the patient was in good general condition, with significant improvement in visual symptoms after surgery and persistent headache as the main residual symptom. No other neurological deficits were observed at baseline assessment.

**Figure 3:**
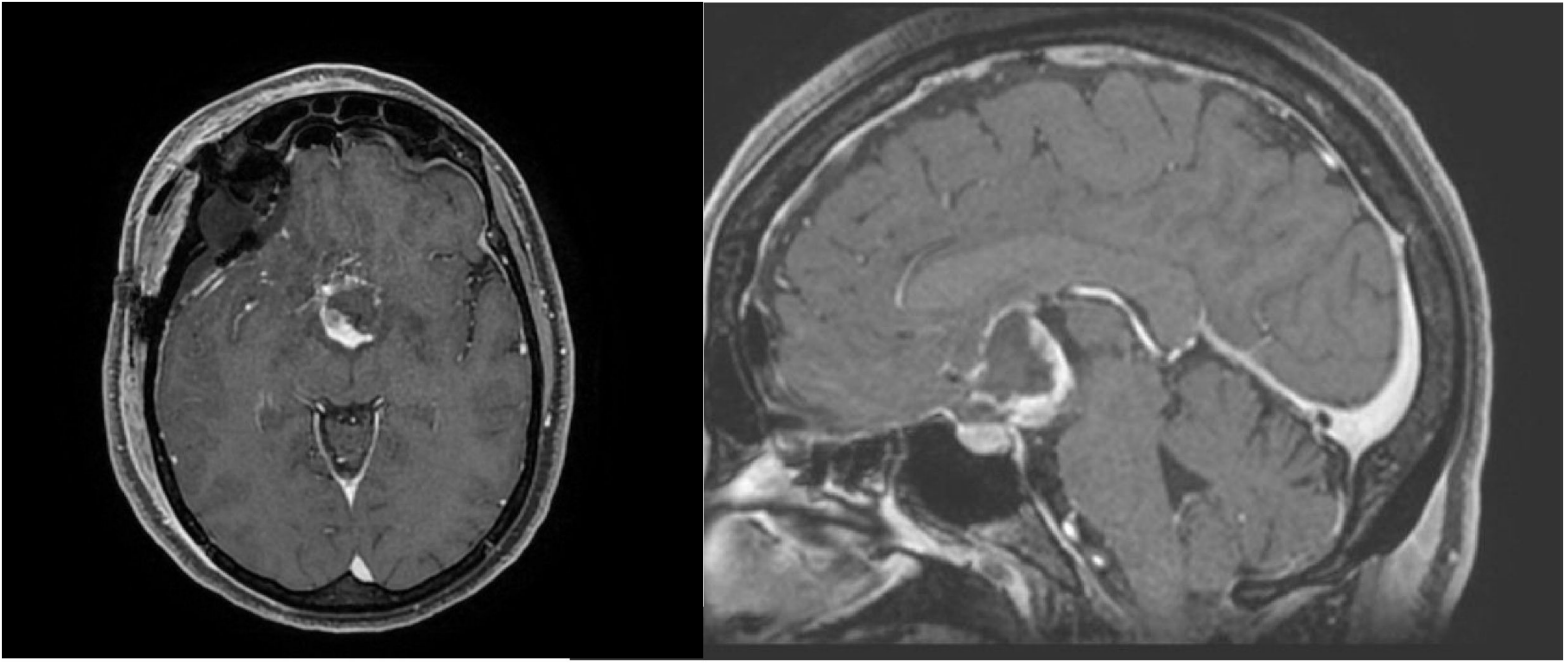
Target volume delineation of the clinical case.

Radiotherapy was performed using Radixact Helical TomoTherapy (Accuray, Sunnyvale, CA, USA), following a conventional fractionation schedule of 59.4 Gy delivered in 33 daily fractions. Target volume delineation was based on T1-weighted contrast-enhanced sequences to define the gross tumor volume (GTV). The planning target volume (PTV) consisted of the GTV plus an isotropic margin of 3 mm. An overlap structure was delineated in the intersection between the PTV and optic chiasm, with a prescription dose of 56.4 Gy to comply with dose constraints for optic structures (**Figure 3**).

Treatment was well tolerated. The patient received prophylactic low-dose steroids (dexamethasone 2 mg/day for the entire treatment duration), and no treatment interruptions occurred. No major side effects were observed, and the headache symptoms resolved. One month after completing radiotherapy, the patient continued steroid therapy for two weeks due to persistent headache, which led to suspected iatrogenic Cushing syndrome that was further investigated by the hospital’s Endocrinology and Rheumatology teams.

Headache was fully resolved at the first follow-up visit, 45 days after the end of treatment. Three months later, the patient developed asthenia and depression; laboratory tests and clinical evaluation led to a diagnosis of systemic sclerosis.

At 12 months of follow-up, serial MRI examinations showed no change in the lesion, indicating stable disease after radiotherapy. The patient remains in good general condition, fully recovered from metabolic disturbances, and is under rheumatologic surveillance, with no major neurological disorders or relevant radiotherapy-related side effects at the last follow-up visit.

### 3.2 Literature review

After the initial search identified 18 studies, additional hand searching of bibliographies yielded a final total of 14 studies (**Table 1**) (1, 9–21).

**Table 1:** Literature experiences of radiotherapy for chordoid glioma.

Author (year)	Patients (n)	Age, gender	Clinical presentation	Tumor size	Surgical approach	Resection extent	Radiotherapy technique	Radiotherapy dose	Clinical outcomes	JBI critical assessment tool
Present Study-Case (2024)	1	41yr, female	Headache, Sight impairment, nausea	29 mm	Right pterional approach	GTR	IMRT-IGRT	59.4 Gy/33 fx	Alive in disease control after 12 months	–
Zhang et al. (2020) (9)	1	43yr, female	Headache, Sight impairment	62 mm	Transcallosal approach	GTR	NR	NR	Alive after 15 months	Low
Danilowicz et al. (2018) (10)	1	18yr, female	Headache, diabetes insipidus, adrenal insufficiency	35 mm	Right pterional approach	GTR	GK	NR	Alive after 3 months	Low
Yang et al. (2017) (11)	1	50yr, female	Sight impairment	41 mm	NR	GTR	GK	11.5 Gy/1 fx	Alive after 16 months	Low
Erwood et al. (2017) (12)	1	46yr, female	Neurocognitive changes, Headache	48 mm	Transcortical transventricular craniotomy	STR	EBRT	45 Gy/25fx + boost up to 9 Gy	Death by disease after 7 months	Moderate
Morais et al. (2015) (13)	1	13yr, female	Diabetes insipidus	NR	NR	STR	EBRT	50.4 Gy/28fx	Disease relapse after 12 months	Moderate
Kobayashi et al.(2013) (14)	2	55yr, female 31yr, female	Headache, nausea Sight impairment	18 mm	Biopsy Biopsy	No resection No resection	GK GK	11 Gy/1fx 10.5 Gy/1fx	Alive in disease control after 70 months Alive in disease control after 66 months	Moderate
Zarghouni et al. (2012) (15)	1	NR	Sight impairment	NR	NR	STR	SRS	NR	Alive with sight recovery at unspecified follow up	Low
Iwami et al. (2009) (16)	1	61yr, female	Syncope	35 mm	Bifrontal Basal interhemispheric approach	GTR	GK	12 Gy/1fx	Alive in disease control after 12 months	Low
Kurian et al. (2005) (17)	2	32yr, female 37yr, female	Headache, endocrine disorders Sight impairment	24 mm NR	Partial Resection Biopsy	GTR No resection	BRT-I^192^ BRT-I^192^	NR NR	Alive in disease control after 15 months Death by recurrence after 9 months	Moderate
Nakajima et al. (2003) (18)	1	49yr, female	Memory impairment, urinary incontinence	40 mm	Bifrontal craniotomy	GTR	GK	15-18Gy/1fx	Alive in disease control after 24 months	Moderate
Hanbali et al. (2001) (19)	1	57yr, male	Headache, Smell impairment	10 mm	Right frontal craniotomy	GTR	EBRT	54 Gy/30fx	Death by other causes 3 weeks after RT	Low
Tonami et al. (2000) (20)	1	42yr, female	Endocrine disorders	NR	Translaminar terminalis	STR	EBRT	52 Gy/26fx plus focal boost 20 Gy	Alive in disease control after 9 months	Low
Reifenberger et al. (1999) (21)	1	56yr, female	Fatigue, headache	23 mm	Transventricular approach	STR	GK	NR	Alive in disease control after 42 months	Low
Brat et al. (1998) (1)	2	31yr, female 59yr, female	Obstructive hydrocephalus, weight loss	NR NR	NR NR	STR STR	NR NR	NR NR	Recurrence after 48 months Death by disease recurrence after 36 months	Low

In total, 18 patients (including the present case) received radiotherapy for chordoid glioma, mainly in the form of Gamma Knife radiosurgery, with doses ranging from 11.5–18 Gy in patients with postoperative residual disease or who were unfit for surgery (five and two patients, respectively). Details regarding the radiotherapy technique were not reported for four patients, whereas brachytherapy with I^192^-seeds was described in two patients in the study by Kurian et al. (17)

Conventional radiotherapy was reported in only five patients, including our case, with doses ranging from 45–59.4 Gy in 1.8–2 Gy per fraction (12, 13, 19, 20).

Most patients treated with adjuvant radiotherapy were female (16 cases), with one male and one unreported, and a median age at diagnosis of 40 years (range, 13–57 years). The most common presenting symptom was headache (44% of cases), followed by visual deficits (13.6%).

Radiotherapy was performed after subtotal resection in 33% of cases and after gross total resection in 44% of cases. The remaining patients received radiotherapy as definitive treatment after stereotactic biopsy.

Three patients died due to disease recurrence at 7, 9, and 36 months, respectively. Recurrence after radiotherapy was also described in two other cases, at 12 and 48 months. In the remaining cases, the median local control time was 26 months. No patient in this series received chemotherapy.

## 4 Discussion

Chordoid glioma is a relatively newly characterized glial neoplasm, first described by Brat et al. as typically located near the third ventricle. From a pathological standpoint, the histogenesis of this tumor remains unclear, although strong GFAP positivity supports a likely glial origin (22).

Fewer than 100 cases have been reported in the literature. Radiologically, chordoid glioma is often difficult to distinguish from craniopharyngioma, as both present as well-defined, T1-isointense, ovoid masses with consistent and homogeneous contrast enhancement. Clinically, presentation is heterogeneous—ranging from incidental, asymptomatic cases to a broad spectrum of symptoms including headache, memory deficits, visual impairment, endocrine or metabolic disorders, and, in some cases, psychosis (23).

Although categorized as a WHO Grade II glioma, the prognosis for chordoid glioma is often poor, primarily due to the low likelihood of achieving complete surgical resection regardless of the surgical approach. The extent of resection remains one of the main predictors of improved survival, as tumor recurrence is more likely when gross total resection is not achieved, with a reported 5-year progression-free survival rate of 35.5%.

Nonetheless, a systematic review by Ampie et al. highlighted that surgical resection remains the cornerstone of treatment, while the role of adjuvant therapy remains controversial. Similarly, Huo et al. emphasized the uncertain benefit of adjuvant therapy for chordoid glioma (24).

However, severe postoperative complications are frequently reported, including cerebrospinal fluid leakage, hypothalamic dysfunction, sinonasal morbidity, and pulmonary thromboembolism. Therefore, several authors have proposed a potential role for adjuvant radiotherapy as a therapeutic strategy that may combine less invasive surgery with improved local disease control (25).

As chemotherapy has not been reported in the literature, radiotherapy remains the most common adjuvant treatment, with a slight preference for radiosurgery—likely due to the tumor’s critical location and the need to minimize exposure of nearby healthy structures.

Some authors have also reported the use of conventional radiotherapy, although with generally poor outcomes, possibly related to the relatively low total doses delivered. In the present case, after multidisciplinary discussion, we decided to perform conventional radiotherapy with the aim of delivering a higher biological dose to the target compared with previously reported schedules, given the unfavorable prognosis of this neoplasm.

Our patient received 59.4 Gy in 33 fractions—the highest conventional radiotherapy dose reported to date—derived from prior institutional experience in treating rare brain tumors. This approach was based on a similar adjuvant radiotherapy schedule applied to a young adult patient with papillary pineal tumor, which achieved favorable results.

After 12 months of follow-up, no evidence of local progression was observed on serial MRI scans, and the patient fully recovered from the initial persistent headache. The overlapping diagnosis of systemic sclerosis immediately after radiotherapy slowed the recovery process but did not affect oncological outcomes, reinforcing limited existing evidence supporting the feasibility of radiotherapy in patients with connective tissue diseases or immunosuppression (26, 27).

Consistently, a recent study by Rzepka et al. (29) assessed chromosomal radiosensitivity in oncological and non-oncological patients, showing higher radiosensitivity rates—defined as the average number of DNA breaks per metaphase—in oncological patients with connective tissue disorders (29).

This study has several limitations, beginning with its case-report design, which inherently restricts the level of evidence. Moreover, the data available in the literature and summarized here remain limited and carry a low level of evidence, being mainly composed of case reports and small case series. Many of these studies also lack key information regarding treatment parameters and clinical outcomes, thereby preventing a direct comparison between stereotactic and conventional radiotherapy approaches.

## 5 Conclusions

Considering the rarity of this disease, the management of chordoid glioma remains controversial. The use of modern adjuvant radiotherapy techniques may represent a favorable option following surgery, potentially reducing the incidence of severe postoperative sequelae and improving local tumor control. Larger databases and national registries with longer follow-up periods are necessary to gain more insights into recurrence patterns and optimal therapeutic strategies.

## Data Availability

The original contributions presented in the study are included in the article/supplementary material. Further inquiries can be directed to the corresponding author.
